# Erkrankungsprävalenzen und Leistungsunfähigkeit von Menschen in Arbeitslosigkeit in Deutschland: Eine Analyse der Daten des Ärztlichen Dienstes 2016–2021

**DOI:** 10.1055/a-2763-8046

**Published:** 2026-01-27

**Authors:** Andreas Guenter Franke, Kirsi Manz, Norbert Scherbaum, Claudia Pieper, Patrik Roser, Gabriele Lotz-Metz

**Affiliations:** 1 Fachbereich Beratungswissenschaften, Hochschule der Bundesagentur für Arbeit, Mannheim; 2 Ärztlicher Dienst, Bundesagentur für Arbeit, Frankfurt am Main; 3 Klinik für Hämatologie, Hämostaseologie, Onkologie und Stammzellentransplantation, Medizinische Hochschule Hannover; 4 LVR-Universitätsklinik Essen, Klinik für Psychiatrie und Psychotherapie, Medizinische Fakultät, Universität Duisburg-Essen, Essen; 5 Institut für Medizinische Informatik, Biometrie und Epidemiologie, Universitätsklinikum Essen; 6 Ärztlicher Dienst, Bundesagentur für Arbeit, Nürnberg

**Keywords:** Arbeitslosigkeit, Leistungsfähigkeit, psychische Erkrankungen, Langzeitarbeitslosigkeit, Epidemiologie, Unemployment, work ability, mental disorders, Epidemiology, long-term Unemployment

## Abstract

**Einleitung:**

Seit langem ist der grundsätzliche Zusammenhang zwischen Arbeitslosigkeit (ALO) und gesundheitlichen Beeinträchtigungen bekannt. Dabei stellen sich Fragen nach den häufigsten Erkrankungen unter Menschen in ALO sowie darauf basierende (etwaige) Leistungseinbußen. Ziel der Studie ist es, die 20 häufigsten Erkrankungen von im Ärztlichen Dienst (ÄD) der Bundesagentur für Arbeit (BA) untersuchten Menschen in ALO darzustellen sowie (etwaige) darauf basierende Leistungseinbußen für den (allgemeinen) Arbeitsmarkt und weitere assoziierte Faktoren zu analysieren.

**Methoden:**

Für die vorliegende mehrjährige Querschnittsstudie (2016–2021) wurden die 20 häufigsten Erkrankungen/ Diagnosen aller (n=4 249 028) sozialmedizinischer Begutachtungen des ÄD der BA mit den jeweiligen Erst- und Zweitdiagnosen auf der Basis von ICD-10-Codes sowie damit assoziierte Faktoren (Geschlecht, Alter, Leistungsbild) aus Datenbanken des ÄD analysiert. Zur Analyse der Daten wurde neben deskriptiver Statistik binäre logistische Regression genutzt.

**Ergebnisse:**

Von den jährlich rund 500 000 im ÄD begutachteten KundInnen erhielten nahezu alle (2016: 90,1%; 2021: 99,5%) eine Erstdiagnose. Von den 20 häufigsten Diagnosen stammt die Hälfte aus dem Bereich der psychischen Erkrankungen. Am häufigsten wurden depressive Episoden (n=416 531; 10,2%), rezidivierend depressive Störungen (n=415 651; 10,1%) und (andere) Angststörungen (n=169 112; 4,6%) diagnostiziert. Bei den nicht-psychiatrischen Störungen belegen Rückenschmerzen (n=243 389; 6,6%), essentielle Hypertonien (n=146 316; 4,0%) und (sonstige) Bandscheibenschäden (n=91 861; 2,2%) die ersten drei Plätze. Alter und Geschlecht differieren je nach Erkrankung signifikant. Insgesamt wurden 33,9% aller begutachteten Personen als leistungsunfähig (<3 Std./Tag) eingestuft, wobei psychische Erkrankungen den häufigsten Grund dafür darstellen, dabei am häufigsten mit 63,6% Schizophrenien.

**Diskussion:**

Psychische Erkrankungen sind unter von ALO betroffenen Menschen besonders häufig. Dies sollte bei der ärztlichen Versorgung sowie der Arbeitsmarktintegration Beachtung finden. Die Vernetzung von arbeitsmarktlichen und gesundheitlichen und insbesondere psychiatrischen Hilfen scheint sinnvoll, um psychische Erkrankungen und ALO möglichst rasch und nachhaltig zu überwinden. Stichworte: Arbeitslosigkeit, Erkrankungen, Epidemiologie, Leistungsunfähigkeit, Langzeitarbeitslosigkeit

## Abstract (en)

**Introduction:**

The fundamental association between unemployment and health problems has long been recognized. This raises questions about the most common illnesses among unemployed people and any resulting loss of productivity. The aim of the study is to present the 20 most common illnesses among unemployed people examined by the Medical Service (ÄD) of the Federal Employment Agency (BA) and to analyze any resulting loss of productivity for the (general) labor market and other associated factors.

**Methods:**

For this multi-year cross-sectional study study (2016–2021), the 20 most common diseases/ diagnoses from all (n=4,249,028) social medical assessments conducted by the Medical Service (ÄD) of the Federal Employment Agency were analyzed, along with the respective primary and secondary diagnoses based on ICD-10 codes and associated factors (gender, age, performance profile) from the ÄD databases. For data analyses descriptive statistics and binary logistic regression were used.

**Results:**

Of the approximately 500,000 social medical assessments carried out by the MS each year, nearly all clients (2016: 90.1%; 2021: 99.5%) received a diagnosis. Depressive episodes (n=416,531; 10.2%), recurrent depressive disorders (n=415,651; 10.1%) and (other) anxiety disorders (n=169,112; 4.6%) belong to the most frequent diagnoses. Among the non-psychiatric disorders, back pain (n=243,389; 6.6%), (primary) hypertension (n=146,316; 4.0%) and (other) disc damage (n=91,861; 2.2%) occupy the top three places. Age and gender differed significantly depending on the disease. Overall, 33.9% of all assessed clients were classified as incapacitated (<3 hours/day), with mental illnesses being the most common reason for this, with schizophrenia being the most common at 63.6%).

**Discussion:**

Mental disorders are particularly common among people who are unemployed. This should be taken into account in medical care and labor market integration to overcome both: unemployment and (psychiatric) illness. Keywords: Unemployment, illnesses, epidemiology, inability to work, long term unemployment

## Einleitung

In den 1930-er Jahren hat die Marienthalstudie erstmals empirisch den Zusammenhang von Arbeitslosigkeit (ALO) und dadurch entstandener negativer psychosozialer Aspekte demonstriert 1 . Dies wurde seitdem durch zahlreiche Studien bestätigt und ergänzt: Dazu zählen u. a. Erkenntnisse über den Zusammenhang von ALO und Diabetes mellitus sowie Adipositas 2 3 , ALO und Krebserkrankungen 4 5 , ALO und Immobilität, ALO und Fatigue sowie Schmerzen (z. B. bei muskuloskelettalen und rheumatoiden Erkrankungen) 6 7 8 9 10 . Das Vorliegen bzw. Wahrnehmen von Schmerzen als weit verbreitetes unspezifisches Symptom mit erheblicher psychischer Modulation gilt als wesentlicher Risikofaktor für Arbeitsplatzabwesenheit 11 , Frühberentung 12 und gesundheits-assoziiertem Arbeitsplatzverlust 13 . Besonders relevant sind m Zusammenhang mit ALO die oft lang andauernden und wechselhaften psychischen Erkrankungen 14 15 16 17 18 19 20 21 : Studien zeigen beispielsweise den Zusammenhang von ALO und Schizophrenien 14 , Depressionen (über 20 Jahren in 30 Ländern) 15 , Suchterkrankungen (legale sowie illegale psychotrope Substanzen in Nordamerika und Europa) 16 sowie dem allgemeinen psychischen Wohlbefinden 19 . Die Krankheitslast ist insgesamt mit der Dauer der ALO verknüpft 16 17 21 22 23 , was für viele psychische Erkrankungen wie z. B. Suchterkrankungen 16 , Depressionen und Angsterkrankungen 17 22 , affektive Störungen und Suizidalität 17 gilt. Eine Longitudinalstudie von Brydsten und Kollegen hat darüber hinaus eindrucksvoll gezeigt, dass auch weitere (psycho-) soziale Faktoren (z. B. hohe ALO-Quote in der Nachbarschaft) eine wichtige Rolle bei der Wahrnehmung eigener somatischer Symptome spielen 24 . Der Zusammenhang zwischen ALO und Erkrankungen ist multifaktoriell, hoch kompliziert und von Wechselwirkungen gekennzeichnet 17 25 26 : Eine wichtige Rolle spielen beispielsweise die Art, Dauer und Schwere der Erkrankung sowie die Erwerbsbiographie der von ALO Betroffenen und vor allem die Dauer der ALO. Es entsteht mitunter ein „Teufelskreis“ bzw. eine gleichsinnige Beziehung zwischen Erkrankung und ALO 16 27 . Umgekehrt weisen Studien auf die protektive Wirkung von Erwerbsarbeit hinsichtlich der psychischen Gesundheit hin 27 28 29 .

In Deutschland liegt die ALO-Quote bundesweit gegenwärtig (Nov. 2025) bei 6,1% (n=2,885 Mio.) 30 ; 2,1% (n=1,0 Mio.) sind kürzer, 3,9% (n=1,8 Mio.) länger als ein Jahr arbeitslos (Langzeitarbeitslosigkeit, LZA) 30 .

Leider betrachten (inter-) nationale Studien ALO und Krankheitslast fast immer getrennt, oder es wird keine Verbindung hergestellt, wie es beispielsweise bei den Global Burden of Disease Studien der Fall ist; hier wird der Faktor Arbeit bzw. die ALO-Quote leider nicht berücksichtigt 31 . Zudem beruht die etwaige Erfassung des Gesundheitszustandes auf den (subjektiven) Angaben von medizinischen Laien.

Die Bundesagentur für Arbeit (BA) ist für die Vermittlung von Menschen in ALO und alle weiteren Dienstleistungen am Arbeitsmarkt zuständig. Oft ist fraglich, ob arbeitslose Menschen wegen Krankhei t nicht arbeiten können. Der Ärztliche Dienst (ÄD) der BA führt dazu jährlich ca. 500 000 sozialmedizinische Begutachtungen von arbeitslosen sogenannten KundInnen der BA durch 32 , da die (Vermittlungs-) Fachkräfte der BA über keine medizinische Expertise verfügen. Dazu stellen sie entsprechende (vorformulierte) Fragen an den ÄD. Die zuständigen Vermittlungsfachkräfte besprechen dann das Procedere einer sozialmedizinischen Begutachtung mit den KundInnen. Nach Aushändigung der nötigen Unterlagen müssen sich die KundInnen im Rahmen ihrer Mitwirkungspflicht gem. § 62 SGB I und § 32 SGB III für eine sozialmedizinische Begutachtung beim ÄD zur Verfügung stellen. Grundlage der sozialmedizinischen Begutachtungen sind das Einverständnis der KundInnen, medizinische Unterlagen (Befunde, Entlassbriefe, etc.) von Behandlern (HausärztInnen, Kliniken, etc.) sowie je nach Einzelfall auch direkte KundInnenkontakte mit dem ÄD.

Die vorhandenen Studien zum Zusammenhang von (psychischen) Erkrankungen und ALO basieren meist auf Befragungen von selbst betroffenen medizinischen Laien zu ihrer Gesundheit und nicht auf objektivierbaren (fach-) ärztlichen Befunden. Darüber hinaus wird in epidemiologischen Studien zu psychischen Erkrankungen der Faktor ALO meist ausgespart.

Ziel dieser Studie ist es, mit den bundesweiten Daten des ÄD auf der Basis von (fach-) ärztlichen Untersuchungen und Diagnosestellungen ausschließlich unter arbeitslosen Personen aus den Jahren 2016–2021 die 20 häufigsten Erkrankungen unter arbeitslosen Personen und die damit assoziierte Leistungs(un)fähigkeit für den allgemeinen Arbeitsmarkt zu ermitteln und diese Personen durch weitere Variablen genauer zu charakterisieren.

## Methoden

### Studiendesign

Alle sozialmedizinischen Begutachtungen (s. o.) dieser retrospektiven und deskriptiven Längsschnittstudie (2016–2021) wurden von erfahrenen (Fach-) ÄrztInnen des Ärztlichen Dienstes (ÄD) durchgeführt, die mindestens eine vierjährige Erfahrung in der klinischen Medizin haben; die überwiegende Mehrheit verfügt über mindestens eine Facharztqualifikation und die Zusatzqualifikation „Sozialmedizin“ 32 .

Die Stichprobe basiert auf allen sozialmedizinischen Begutachtungen (n=4 249 028) des ÄD der Bundesagentur für Arbeit (BA) der Jahre 2016 bis 2021. Einziges Einschlusskriterium war eine sozialmedizinische Begutachtung im ÄD der BA in den Jahren 2016 bis 2021; es wurden keine Ausschlusskriterien und keine Auswahlverfahren angewendet.

### Studienablauf

Die folgenden Daten wurden in der BA aus den Datenbanken des ÄD (COMEDSQL, Microsoft SQL Server 2016) extrahiert: soziodemographische Daten Alter (in 5-Jahresgruppen), Geschlecht, ein bis zwei Diagnosen (sofern vorhanden erste Diagnose und sofern vorhanden zweite Diagnose) gemäß ICD-10, Angaben zur quantitativen Leistungsfähigkeit der KundInnen (<3 Stunden, 3–6 Stunden,>6 Stunden täglich, keine Angabe), auftraggebende Abteilung innerhalb der BA (Arbeitsvermittlung (AV), Leistung gewährende Abteilung, Jobcenter (JC), Rehaabteilung (Reha), „U25“ (Berufsberatung vor dem Arbeitsleben/ Personen unter dem 25. Lebensjahr), Jahr der Begutachtung. Die Kategorie „keine Angabe“ bei der quantitativen Leistungsfähigkeit stellte eine gültige Antwortkategorie in der Datenbank dar.

Die o.g. Variablen wurden durch die Gutachter in das ÄD- bzw. BA-interne Programm „Comed“ eingegeben und von Comed in einer Access-Datenbank abgelegt.

Die anonymisierten Daten wurden nach Genehmigung der Studie im Oktober 2023 dem Studienverantwortlichen und der Studienstatistikerin von der BA als verschlüsselte Excel-Dateien auf gesicherten Datenträgern zur Verfügung gestellt. Im Anschluss (ab November 2023) daran wurden die Daten entschlüsselt und in die Software „R“ (Version 4.3.2) eingelesen und dort verarbeitet.

### Statistische Analyse

Für die vorliegenden Fragestellungen wurden kontinuierliche Variablen mit Mittelwerten (MW) und Standardabweichungen (SD) angegeben und kategoriale Variablen mit Frequenzen und Prozentwerten. Die 20 häufigsten Diagnosen wurden deskriptiv analysiert; in einem zweiten Schritt wurden statistische Verfahren angewendet: Die Odds ratio (OR) mit dem 95% Konfidenzintervall (KI) für das Vorliegen einer vollen Leistungsfähigkeit wurde mittels binärer logistischer Regressionsanalyse für somatische Diagnosen vs. psychische Diagnosen berechnet. Dabei wurde eine volle Leistungsfähigkeit als>6 Stunden Arbeiten täglich definiert und alle ICD-10 „F-Diagnosen“ als psychische und alle anderen Diagnosen als „somatische“ Diagnosen betrachtet.

### Ethische und datenschutzrechtliche Aspekte

Die Studie enthält keinerlei Daten aus Versuchen mit Tieren und/ oder Menschen und enthält zudem keinerlei personenbeziehbare Daten. Sie wurde unter Beachtung des Datenschutzes gemäß des Bundesbeauftragten für den Datenschutz und die Informationsfreiheit (BfDI) durchgeführt und wurde seitens der Abteilung für Datenschutz der BA eingehend geprüft und im Oktober 2023 genehmigt (No. AZ1400.12-1/2023).

Die Nutzung von Künstlicher Intelligenz fand weder bei der Generierung der Daten noch bei Erstellung des Manuskriptes statt.

## Ergebnisse

### Soziodemographie

In der Datenbank des Ärztlichen Dienstes (ÄD) sind in den Jahren 2016 bis 2021 insgesamt 4 249 028 Datensätze über sozialmedizinische Begutachtungen mit und ohne KundInnenkontakt enthalten. Die KundInnen sind im statistischen Mittel 43,3 Jahre alt (SD: 13,2 Jahre); 52,4% der KundInnen sind männlich; Daten zu ausschließlich psychischen Erkrankungen sowie Diagnosegruppen (ICD-Kapitel) wurden bereits separat veröffentlicht 33 34 .

Bei den meisten begutachteten KundInnen (n=4 100 402, 96,5%) wurde bei der Begutachtung im ÄD mindestens eine Diagnose gestellt ( Tab. 1 ). Nur 3,5% (n=148 626) erhielten keine Diagnose. Der Anteil der begutachteten KundInnen mit mind. einer Diagnose stieg über den Zeitraum 2016–2021 deutlich an ( Tab. 1 ).

**Table:** **Tab. 1** Diagnosehäufigkeit

Jahr	Erste Diagnose in% (n)	Zweite Diagnose in% (n)
2016	90,1% (528 970)	76,3% (448 127)
2017	95,3% (603 564)	85,2% (539 629)
2018	95,6% (644 740)	86,5% (583 591)
2019	97,4% (803 507)	87,5% (722 300)
2020	99,3% (785 273)	89,8% (710 005)
2021	99,5% (734 348)	91,2% (673 488)
*2016-2021*	*96,5% (4 100 402)*	86,5% ( *3.677 140)*

Berechnungsgrundlage ist die Gesamtanzahl der im jeweiligen Jahr durchgeführten sozialmedizinischen Begutachtungen mit und ohne KundInnenkontakt, n=4 249 028; n=Anzahl

### Diagnosehäufigkeiten

Hinsichtlich der Erstdiagnosen war die häufigste Diagnose mit 10,2% eine depressive Episode (ICD-10: F32) gefolgt von rezidivierend depressiven Störungen (ICD-10: F33) mit 10,1% und Rückenschmerzen (ICD-10: M54) mit 6,3% der Erstdiagnosen. Auf Platz vier lagen mit 3,5% Reaktionen auf schwere Belastungen und Anpassungsstörungen (ICD-10: F43) und auf Platz fünf mit 3,5% (andere) Angststörungen (ICD-10: F41).

Hinsichtlich der Zweitdiagnosen waren die fünf häufigsten Diagnosen Rückenschmerzen (ICD-10: M54) mit 6,6%, depressive Episoden (ICD-10: F32) mit 4,8% und (andere) Angststörungen (ICD-10: F41) mit 4,6% sowie an Platz vier und fünf somatoforme Störungen (ICD-10: F45) mit 4,3% und Reaktionen auf schwere Belastungen und Anpassungsstörungen (ICD-10: F43) mit 4,0%.

Die übrigen der 20 häufigsten Diagnosen ergeben sich aus Tab. 2 ; weniger häufig als die 20 häufigsten Diagnosen sind sowohl hinsichtlich der Erst- als auch hinsichtlich der Zweitdiagnosen seltener als 1% und werden daher weder im Text noch in den Tabellen aufgelistet.

**Table:** **Tab. 2** Häufigkeitsverteilung der Diagnosen.

Rang	1. Diagnose, ICD-10-Schlüssel (%, n)	2. Diagnose, ICD-10-Schlüssel (%, n)
1.	Depressive Episode (F32) (10,2%, 416 531)	Rückenschmerzen (M54) (6,6%, 243 389)
2.	Rezidivierend depressive Strg. (F33) (10,1%, 415 651)	Depressive Episode (F32) (4,8%, 175 903)
3.	Rückenschmerzen (M54) (6,3%, 257 167)	(Andere) Angststrg. (F41) (4,6%, 169 112)
4.	Reaktionen schwere Belastungen und Anpassungsstrg. (F43) (3,5%, 144 833)	Somatoforme Strg. (F45) (4,3%, 156 614)
5.	(Andere) Angststrg. (F41) (3,5%, 142 282)	Reaktionen schwere Belastungen und Anpassungsstrg. (F43) (4,0%, 147 399)
6.	Somatoforme Strg. (F45) (3,0%, 124 010)	Essentielle (prim.) Hypertonie (I10) (4,0%, 146 316)
7.	Psych. und Verhaltensstrg. durch Alkohol (F10) (2,8%, 113 150)	Rezidivierend depressive Strg. (F33) (3,6%, 132 496)
8.	(Sonstige) Bandscheibenschäden (M51) (2,2%, 91 861)	Spez. Persönlichkeitsstrg. (F60) (2,4%, 88 761)
9.	Schizophrenie (F20) (1,9%, 79 094)	Psych. und Verhaltensstrg. durch Alkohol (F10) (2,1%, 75 297)
10.	Gonarthrose (M17) (1,9%, 76 814)	Phobische Strg. (F40) (2,0%, 73 885)
11.	Spez. Persönlichkeitsstrg. (F60) (1,8%, 74 977)	Spondylose (M47) (1,9%, 71 437)
12.	Spondylose (M47) (1,8%, 74 679)	(Sonstige) Bandscheibenschäden (M51): (1,9%, 70 965)
13	Chron. ischämische Herzkrankheit (I25) (1,6%, 65 629)	Adipositas (E66) (1,8%, 65 157)
14.	Schulterläsionen (M75) (1,3%, 51 723)	Gonarthrose (M17) (1,6%, 57 280)
15.	Psych. und Verhaltensstrg. durch multiplen Substanzgebrauch (F19) (1,2%, 47 534)	Sonstige Gelenkkrankheiten (a.n.k., M25) (1,4%, 52 619)
16.	Phobische Strg. (F40) (1,2%, 46 986)	Diabetes mellitus (Typ 2) (E11) (1,4%, 50 047)
17.	Schmerz (a.n.k.) (R52) (1,1%, 45 862)	Schulterläsionen (M75) (1,3%, 46 773)
18.	Sonstige chron. obstruktive Lungenkrankheit (J44) (1,1%, 45 773)	Sonstige Krankheiten der Wirbelsäule und des Rückens (a.n.k.) (M53) (1,3%, 46 425)
19.	Sonstige Krankheiten der WS und des Rückens (a.n.k.) (M53) (1,1%, 43 619)	Chron. ischämische Herzkrankheit (I25): (1,3%, 46 135)
20.	Essentielle (prim.) Hypertonie (I10) (1,1%, 43 579)	Psych. und Verhaltensstrg. durch Cannabinoide (F12) (1,2%, 42 579)

a.n.k.=andernorts nicht klassifiziert; Strg.=Störung, WS=Wirbelsäule

Psychiatrische Diagnosen sind unter den 20 häufigsten Erst- und Zweitdiagnosen mit rund 50% vertreten; Daten zu psychiatrischen Diagnosegruppen wurden separat publiziert 34 .

### Alter bei Diagnosestellung im ÄD

Das Alter der KundInnen unterscheidet sich je nach Diagnose. Das niedrigste Alter mit 29,8 Jahren liegt bei den Persönlichkeitsstörungen, Schizophrenien (32,6 Jahre) sowie Cannabiskonsumstörung (33,5 Jahre).

### Geschlechtsunterschiede

Die größten Geschlechtsunterschiede der KundInnen mit den 20 häufigsten Diagnosen liegen bei der Erstdiagnose bei den chronisch-ischämischen Herzkrankheiten (83,3% männlich) und den Persönlichkeitsstörungen (34,2% männlich) sowie den Zweitdiagnosen Cannabiskonsumstörungen (81,3% männlich) und somatoformen Störungen (36,2% männlich). Weitere Angaben sind Tab. 3 zu entnehmen.

**Table:** **Tab. 3** Diagnose in Relation zu Alter und Geschlecht der begutachteten KundInnen.

	1. Diagn. (ICD-10)	Alter MW	Männl. Geschlecht% (n)	2. Diagn. (ICD-10)	Alter MW	Männl. Geschlecht% (n)
1.	F32	43,8	43,1% (179 668)	M54	47,5	49,7% (120 938)
2.	F33	43,7	39,2% (162 800)	F32	42,3	44,8% (78 802)
3.	M54	46,6	55,7% (143 315)	F41	42,5	38,3% (64 825)
4.	F43	40,8	38,9% (56 294)	F45	46,3	36,2% (56 648)
5.	F41	41,3	39,7% (56 485)	F43	41,2	36,9% (54 409)
6.	F45	46,5	37,5% (46 466)	I10	50,8	59,9% (87 676)
7.	F10	45,9	79,4% (89 881)	F33	41,0	40,1% (53 177)
8.	M51	45,2	61,1% (56 101)	F60	33,9	40,8% (36 231)
9.	F20	32,6	70,4% (55 658)	F10	44,1	75,7% (57 007)
10.	M17	52,5	49,9% (38 288)	F40	36,4	45,5% (33 585)
11.	F60	29,8	34,2% (25 649)	M47	50,8	51,3% (36 617)
12.	M47	50,1	55,3% (41 285)	M51	47,1	57,2% (40 566)
13.	I25	53,3	83,3% (54 660)	E66	41,0	50,2% (32 728)
14.	M75	50,4	57,1% (29 522)	M17	52,3	49,6% (28 425)
15.	F19	35,3	79,4% (37 718)	M25	46,2	54,4% (28 596)
16.	F40	33,5	48,2% (22 649)	E11	51,1	62,4% (31 207)
17.	R52	48,5	44,6% (20 470)	M75	50,8	55,4% (25 907)
18.	J44	53,2	62,7% (28 704)	M53	48,7	43,8% (20 327)
19.	M53	48,3	46,7% (20 377)	I25	53,5	81,2% (37 470)
20.	I10	50,4	58,0% (25 261)	F12	30,1	81,3% (34 619)

ICD-Aufschlüsselung siehe Tab. 2 .

### Quantitative Leistungs(un)fähigkeit

Die für die KundInnen sowie den Arbeitsmarkt entscheidende Frage nach der Leistungsfähigkeit (vollständige quantitative Leistungsminderung=Leistungsunfähigkeit=Leistungsfähigkeit<3 Std. tgl.; Teilleistungsfähigkeit 3–6 Std. tgl.; volle quantitative Leistungsfähigkeit>6 Std. tgl.) in Abhängigkeit von der jeweiligen Diagnose ist in Tab. 4 dargestellt. Insbesondere Personen mit Schizophrenien sind leistungsunfähig (63,6%). Darüber hinaus besteht Leistungsunfähigkeit v. a. bei Polytoxikomanien (51,1%) und psychischen sowie Verhaltensstörungen durch Alkohol (48,1%). Am seltensten wurde eine Leistungsunfähigkeit bei somatischen Erkrankungen festgestellt (z. B. Rückenschmerzen 10,9%). Eine vollständige Leistungsfähigkeit (>6 Std. tgl.) wurde ebenfalls überwiegend bei somatischen Erkrankungen festgestellt (z. B. Spondylosen 69,5%, Schulterläsionen 66,2%, etc. Teilleistungsfähigkeiten (3–6 Std. tgl.) sind die Ausnahme.

**Table:** **Tab. 4** Leistungsfähigkeit der begutachteten KundInnen mit den 20 häufigsten Diagnosen.

	1. Diagn. (ICD-10)	<3 Std.% (n)	3–6 Std.% (n)	>6 Std.% (n)	Keine Angabe% (n)
1.	F32	44,7% (186 168)	3,8% (15 923)	31,9% (132 711)	19,6% (81 729)
2.	F33	44,3% (184 184)	5,3% (22 204)	33,8% (140 576)	16,5% (68 687)
3.	M54	10,9% (27 940)	3,2% (8143)	61,9% (159 270)	24,0% (61 814)
4.	F43	40,2% (58 280)	4,8% (6874)	35,5% (51 427)	19,5% (28 252)
5.	F41	42,8% (60 927)	5,8% (8301)	31,4% (44 703)	19,9% (28 351)
6.	F45	32,1% (39 842)	6,5% (8103)	41,2% (51 123)	20,1% (24 942)
7.	F10	48,1% (54 432)	4,7% (5295)	26,6% (30 073)	20,6% (23 350)
8.	M51	14,3% (13 135)	2,5% (2333)	63,8% (58 624)	19,3% (17 769)
9.	F20	63,6% (50 294)	5,3% (4220)	15,1% (11 933)	16,0% (12 647)
10.	M17	14,6% (11 182)	3,1% (2346)	61,3% (47 045)	21,1% (16 241)
11.	F60	46,8% (35 121)	6,2% (4665)	28,5% (21 379)	18,4% (13 812)
12.	M47	12,4% (9237)	4,1% (3063)	69,5% (51 911)	14,0% (10 468)
13	I25	21,3% (13 957)	4,6% (3030)	52,1% (34 177)	22,0% (14 465)
14.	M75	12,2% (6331)	1,9% (959)	66,2% (34 219)	19,8% (10 214)
15.	F19	51,1% (24 286)	6,6% (3130)	19,5% (9262)	22,8% (10 856)
16.	F40	45,8% (21 535)	5,6% (2629)	29,9% (14 025)	18,7% (8797)
17.	R52	36,6% (16 794)	6,0% (2743)	40,6% (18 611)	16,8% (7714)
18.	J44	30,1% (13 790)	5,4% (2489)	42,0% (19 222)	22,4% (10 272)
19.	M53	11,4% (4968)	3,3% (1452)	61,5% (26 834)	23,8% (10 365)
20.	I10	15,5% (6768)	4,3% (1852)	55,1% (24 014)	25,1% (10 945)

ICD-Aufschlüsselung siehe Tab. 2 . Prozente sind pro Diagnose angegeben.

Die Odds für das Vorliegen einer vollen Leistungsfähigkeit sind für somatische Diagnosen um den Faktor 3,2 höher als bei psychischen Diagnosen (OR=3,18, KI: 3,16–3,20).

### Auftraggebende Instanzen

Bei den Auftraggebenden der Begutachtungen zeigte sich sowohl für die Jobcenter (JC), Arbeitsvermittlung (AV) als auch für U25 (unter 25-Jährige), dass die beiden am häufigsten gestellten Diagnosen pro Auftraggebenden depressive Episoden und rezidivierende depressive Störungen waren. An dritter Stelle waren bei den langzeitarbeitslosen (LZA) KundInnen (JC als Auftraggeber) psychische und Verhaltensstörungen durch Alkohol, bei KundInnen mit Auftrag aus der Arbeitsvermittlung waren es Rückenschmerzen und im Bereich U25 spezielle Persönlichkeitsstörungen.

In einer Subgruppenanalyse der Daten der KundInnen aus der Arbeitsvermittlung (AV), aus JC und U25 wurde die Leistungsfähigkeit untersucht (siehe Tab. 5 ). Es zeigt sich, dass v. a. LZA-KundInnen der JC mit 46,4% besonders häufig leistungsunfähig (<3 Std. tgl.) sind, während die KundInnen der „normalen“ AV deutlich seltener leistungsunfähig sind (29,5%). Im Bereiche U25 wurde rund 1/3 als leistungsunfähig (35,3%) und ca. 42% als voll leistungsfähig eingestuft (42,0%).

**Table:** **Tab. 5** Auftraggebende im Kontext zur Leistungsfähigkeit der begutachteten KundInnen.

Leistungsfähigkeit	Arbeitsvermittlung% (n)	Jobcenter% (n)	U25% (n)
<3 Std.	29,5% (417 835)	46,4% (152 515)	35,3% (13 630)
3–6 Std.	5,3% (74 558)	6,8% (22 378)	3,4% (1 311)
>6 Std.	45,7% (647 314)	23,1% (75 741)	42% (16 195)
Keine Angabe	19,5% (275 769)	23,7% (78 025)	19,3% (7 441)

U25=Berufsberatung vor dem Arbeitsleben / Unter 25-Jährige. Prozente sind spaltenweise berechnet und stellen den Anteil der Leistungsfähigkeit pro Auftraggebenden dar. Daten werden für die 1 782 712 Aufträge aus den genannten Abteilungen gezeigt

## Diskussion

Ziel der Studie war es, die 20 häufigsten Diagnosen der im Ärztlichen Dienst (ÄD) begutachteten KundInnen sowie die damit assoziierten Einbußen der Leistungsfähigkeit darzustellen und damit assoziierte Variablen zu analysieren. Die vorliegende Studie hat gezeigt, dass über 90% der beim ÄD der Bundesagentur für Arbeit (BA) sozialmedizinisch begutachteten KundInnen mindestens eine Erkrankung aufweisen; die drei häufigsten Diagnosen sind depressive Störungen, Rückenschmerzen und Angststörungen. Rund die Hälfte der 20 häufigsten Erkrankungen, kommen aus dem Bereich der psychischen Erkrankungen. Alter und Geschlecht variieren genau wie die auf Grund der jeweiligen Erkrankung bestehenden (quantitativen) Leistungseinbußen je nach Erkrankung. Leistungsunfähigkeiten ergeben sich v. a. bei KundInnen mit psychischen Erkrankungen sowie langzeitarbeitslosen (LZA) KundInnen.

Zunächst ist die Zunahme der Diagnosehäufigkeit der im ÄD erhobenen Daten zwischen 2016 und 2021 (siehe Tab. 1 ) zu beachten. Dies gilt somit nur für die von Arbeitslosigkeit betroffenen Menschen. Als Referenzdaten dienen die Global Burden of Disease (GBD) Studien, die unter anderem darauf abzielen, die gesundheitlichen Auswirkungen von Krankheiten zu quantifizieren. Die auf Deutschland bezogenen GBD-Daten haben gezeigt, dass zumindest zwischen 1990 und 2010 die DALYs (Disability Adjusted Life Years; Maß für die gesundheitliche Belastung einer Bevölkerung durch die Ermittlung von Jahren, die ein Mensch belastet durch eine Erkrankung lebte oder an ihr starb) insgesamt um 7,3% rückläufig waren 35 . Somit nahm die Krankheitslast in der gesamten Bevölkerung ab, während sie unter den von Arbeitslosigkeit Betroffenen zunahm. In den GBD-/ DALY-Studien wird der Erwerbstätigkeitsstatus allerdings nicht monitoriert, so dass dieser Befund darauf hinweisen könnte, dass die Krankheitslast unter von Arbeitslosigkeit betroffenen Menschen im Vergleich zur Allgemeinbevölkerung signifikant zugenommen hat.

Die GBD-Studien zu DALYs zeigen trotz der o.g. Schwäche ähnliche Erkrankungen auf den ersten vier Plätzen: 1. Herz-Kreislauf-Erkrankungen (19,4%), 2. bösartigen Neubildungen (17,9%), 3. muskuloskelettale Erkrankungen (15,8%) sowie 4. psychische und Verhaltensstörungen (11,4%) 35 36 . Diese Erkrankungen liegen auch unter den begutachteten KundInnen abgesehen von Neoplasien auf den ersten Plätzen, wohingegen aber die somatischen Erkrankungen hinter den psychischen Erkrankungen rangieren.

Bei genauerer Betrachtung sind die häufigsten Ursachen für „Years lived with diseases“ (YLD; Jahre, die ein Mensch durch eine Erkrankung belastet lebt) der GBD-Studien aber psychische Erkrankungen (1. Depressionen, 2. Angststörungen, 3. Schizophrenien) 37 . Dies entspricht den Ergebnissen der hier vorliegenden Studie durchaus und bestätigt Ergebnisse epidemiologischer Studien, die zeigen, dass in Deutschland eine 12-Monats-Prävalenz von 9,3% für affektive Störungen vorliegt; noch häufiger sind allerdings Angststörungen (15,3%) 38 . Frühere Studien haben gezeigt, dass von Arbeitslosigkeit betroffene Menschen ein doppelt so hohes Risiko für Depressionen und Angststörungen verglichen zu Berufstätigen haben 22 39 , was unseren Studienergebnissen entspricht. Aktuelle deutsche Studien in Zusammenarbeit mit zwei Jobcentern (JC) weisen auf Prävalenzraten von 61% für affektive und 58% für Angststörungen hin 40 41 .

Welt- und v. a. europaweit gab es zwischen 1990 und 2019 eine überproportionale Zunahme psychischer Erkrankungen, die insbesondere Personen im erwerbsfähigen Alter betrifft 37 ; dies wiederum entspricht den hohen Prävalenzraten der psychiatrischen Diagnosen in dieser Untersuchung ( Tab. 2 ) und korrespondiert auch mit Daten weiterer Studien der Allgemeinbevölkerung: Nach dem jüngsten Mental Health Survey (groß angelegte Befragung/en teilweise unter Nutzung standardisierter Erhebungsinstrumente zum Vorliegen von psychischen Krankheiten) der Weltgesundheitsorganisation (WHO) leiden ca. 970 Mio. Menschen weltweit unter psychischen Erkrankungen, was ca. 13% der Weltbevölkerung entspricht 42 43 . Der aktuellste Survey unter 6000 Probanden im Rahmen der GEDA-Studien (seit 2008 vom Robert-Koch-Institut durchgeführte repräsentative Bevölkerungsstudien zur GEsundheit in Deutschland Aktuell) ergab eine Prävalenz von depressiven Symptomen während der letzten zwei Wochen von 8,3% in Deutschland 44 . Diese wie auch andere bevölkerungsbasierte Untersuchungen (wie GBD und GEDA) basieren allerdings auf Befragungen von medizinischen Laien; zudem wird in solchen Studien der Erwerbsstatus (meist) nicht monitoriert. Stärken dieser Studie sind also die validere Diagnostik und die Verknüpfung von Gesundheitsdaten mit Daten zum Erwerbsstatus.

Die Ergebnisse des jüngsten National Health Interview Surveys (Centers for Disease Control and Prevention, USA) unterscheiden zumindest teilweise zwischen erwerbstätigen und nicht-erwerbstägigen Personen, wobei Letztere signifikant erhöhte Prävalenzen von Sorgen, Nervosität, Ängsten sowie Schmerzen aufweisen 45 . Dies bestätigt die hier vorliegenden Studienergebnisse, bei denen sehr viele mindestens eine psychische Erkrankung und/ oder Schmerzen im Sinne einer muskuloskelettalen Erkrankung oder bedingt durch andere häufige Erkrankungen (Gonarthrose, Spondylose, etc.) aufwiesen.

Es dürfte nicht verwundern, dass Schmerzen auch in der hier vorliegenden Studie besonders häufig diagnostiziert werden (Platz 1 der 2. Diagnosen), da anhaltenden Schmerzen ungeachtet des Erwerbsstatus bei ca. 10 bis 15% der Menschen zu beobachten sind 46 . Das Vorliegen von (chronischen) Schmerzen unabhängig von der Ursache gilt seit langem als mit ALO eng assoziiert; schließlich sind psychosoziale Faktoren, zu denen auch die ALO sowie psychische Erkrankungen gehören, ein wesentlicher Einflussfaktor von Schmerz(wahrnehmungen) 47 48 49 50 51 .

Dass sich Alter und Geschlecht der erkrankten KundInnen krankheitsspezifisch unterscheiden, dürfte nicht verwundern, da dies auch unabhängig vom Erwerbsfähigkeitsstatus der Fall ist. Beispielsweise liegt das Ersterkrankungsalter bei Schizophrenien zwischen 15–35 Jahren, bei Alkoholabhängigkeiten dominiert das männliche Geschlecht 52 .

Als Folge der ALO führen v. a. psychiatrische Diagnosen zu (quantitativer) Leistungsunfähigkeit (<3 Std. tgl.) wohingegen die Muskel- und Skeletterkrankungen sowie andere somatischen Erkrankungen eher zu einem vollen Leistungsvermögen führen. Gerade bei LZA ist die Belastung durch psychische Erkrankungen überrepräsentiert. Symptome der so häufigen Depressionen und Angst- sowie Panikstörungen wie fehlender Antrieb und/ oder die übermäßige Angst dürfte Betroffene daran hindern, überhaupt am Arbeitspatz erscheinen zu können.

In Bezug auf die Leistungsfähigkeit dürfte es nicht verwundern, dass v. a. bei jüngeren KundInnen (U25) im Vergleich zu anderen KundInnen weniger somatische, sondern vielmehr psychische Erkrankungen vorliegen und deutlich mehr KundInnen in diesem Altersbereich als voll leistungsfähig eingestuft wurden. Die Prävalenzraten und Anzahl somatischer Erkrankungen sowie deren Auswirkungen auf die Leistungsfähigkeit nimmt mit zunehmendem Alter zu.

Es ist darauf hinzuweisen, dass zwar die Verknüpfung von ALO und gesundheitlichen, v. a. psychischen Einbußen, eindeutig ist, dennoch ist die kausale Verknüpfung kompliziert und kann mit dieser Studie nicht aufgeklärt werden. Der Zusammenhang ist eine komplizierte, multifaktorielle (sozial, ökonomisch, psychologisch, bildungspolitisch, etc.), gleichsinnige Beziehung, bei der sowohl ALO zur Zunahme von (psych.) Erkrankungen führt, aber das Vorliegen und die Verschlechterung bereits bestehender (psych.) Erkrankungen auch zu ALO führen z. B. 17 25 26 .

Vor diesem Hintergrund zeigen sich wichtige praktische und gesundheitspolitische Implikationen: Es scheint wichtig, ALO rasch zu durchbrechen oder besser noch gar nicht erst entstehen zu lassen, um eine etwaige damit assoziierte Krankheitsentstehung zu verhüten. Auf der anderen Seite sollten (psych.) Erkrankungen schnellst möglich erkannt und behandelt werden, um die Gefahr für einen krankheitsbedingten Arbeitsplatzverlust zu reduzierten. Der o.g. Teufelskreis bzw. die gleichsinnige Beziehung zwischen ALO und psychischen Erkrankungen 16 27 muss unbedingt vermieden werden, da dieser zu weitereichenden negativen Konsequenzen auf Seiten der ALO und auf Seiten der psychischen Gesundheit führt.

### Limitationen

Das Studiendesign weist zunächst die Stärke einer mehrjährigen Querschnittsstudie (2016 – 2021) sowie die Einbeziehung aller sozialmedizinischer Untersuchungen (100%=4 249 028 Datensätze) auf, so dass die Daten auf der Grundgesamtheit der untersuchten arbeitslos gemeldeten Personen basiert und nicht auf einer (selektierten) Stichprobe. Allerdings findet bereits unter den Auftraggebenden eine Selektion statt, so dass nur Personen begutachtet werden, bei denen durch die Vermittlungsfachkräfte bereits ein Verdacht auf eine etwaige Erkrankung besteht. Es besteht somit in diesem Punkt ein wichtiger Bias und keine Repräsentativität der Daten für alle von ALO betroffenen Personen.

Eine Stärke, die aber zu einer limitierten Vergleichbarkeit zu anderen Studien führt, ist wiederum die Datenbasis der Studie: Die Daten (Diagnosen, Leistungsbild) basieren auf (sozial-) medizinischer Expertise von ÄrztInnen und nicht auf subjektiven Angaben von selbst betroffenen medizinischen Laien, wie es in vielen anderen Studien der Fall ist, die vor allem auf Befragungen basieren.

Hinsichtlich der Erhebungsinstrumente ist anzumerken, dass sozialmedizinische Untersuchungen im Vergleich zu anderen Untersuchungen in der Medizin einen eher geringen Standardisierungsgrad aufweisen. Zudem finden die Begutachtungen beim ÄD der BA teilweise mit und teilweise ohne KundInnenkontakt statt, was wiederum zu Verzerrungen führen könnte.

Ergänzend ist darauf hinzuweisen, dass nur 1. und 2. Diagnose in den entsprechenden Eingabefeldern des Comed eingetragen werden können. Etwaige weitere Diagnosen können in Freitextfeldern enthalten sein, die jedoch im Rahmen dieser Studie nicht ausgewertet werden konnten.

Ein „Störfaktor“ ist sicherlich die COVID-19-Pandemie. Die Kontaktverbote mit reduzierter Begutachtungsanzahl ab 2020 und die anders gelagerte Diagnosehäufigkeit von Atemwegsinfekten und anderen Erkrankungen macht es schwierig, die Jahre vor 2020 mit den Jahren ab 2020 zu vergleichen.

## Fazit für die Praxis

Auf der Basis der Studiendaten scheint es von entscheidender Bedeutung, ALO rasch zu durchbrechen oder besser noch gar nicht erst entstehen zu lassen, um eine etwaige damit assoziierte Krankheitsentstehung zu verhüten. Auf der anderen Seite sollten psychische Erkrankungen schnellst möglich erkannt und behandelt werden, um die Gefahr für einen krankheitsbedingten Arbeitsplatzverlust zu reduzierten.

Der o.g. „Teufelskreis“ bzw. die gleichsinnige Beziehung zwischen ALO und psychischen Erkrankungen 16 27 muss unbedingt vermieden werden, da dieser zu weitereichenden negativen Konsequenzen auf Seiten der ALO und auf Seiten der psychischen Gesundheit führt.

Effektiver im Sinne einer Spezialprävention wäre sicherlich eine Risikostratifizierung, um individuelle Risiken für die Entstehung von psychischen Krankheiten und ALO frühzeitig erkennen zu können. Dafür wäre aber die Verknüpfung der bereits analysierten Daten mit weiteren Gesundheitsdaten sowie erwerbsbiographischen und weiteren Daten (Bildungsbiographie, medizinische Vorgeschichte, etc.) der von ALO betroffenen Personen wichtig.
